# Targeted gene therapy for cancer: the impact of microRNA multipotentiality

**DOI:** 10.1007/s12032-024-02450-1

**Published:** 2024-08-01

**Authors:** Nourhan A. Abou Madawi, Zeinab E. Darwish, Enas M. Omar

**Affiliations:** Oral Pathology Department, Faculty of Dentistry, Alexandria University, Champollion Street, Azarita, 21521 Alexandria Egypt

**Keywords:** Cancer, Targeted therapy, Gene therapy, Gene expression, RNA interference, MicroRNA

## Abstract

Cancer is a life-threatening disease and its management is difficult due to its complex nature. Cancer is characterized by genomic instability and tumor-associated inflammation of the supporting stoma. With the advances in omics science, a treatment strategy for cancer has emerged, which is based on targeting cancer-driving molecules, known as targeted therapy. Gene therapy, a form of targeted therapy, is the introduction of nucleic acids into living cells to replace a defective gene, promote or repress gene expression to treat a disease. MicroRNAs (miRNAs) are non-coding RNAs (ncRNAs) that regulate gene expression and thus are involved in physiological processes like cell proliferation, differentiation, and cell death. miRNAs control the actions of many genes. They are deregulated in cancer and their abnormal expression influences genetic and epigenetic alterations inducing carcinogenesis. In this review, we will explain the role of miRNAs in normal and abnormal gene expression and their usefulness in monitoring cancer patients. Besides, we will discuss miRNA-based therapy as a method of gene therapy and its impact on the success of cancer management.

## Introduction

Cancer is a lethal disease. Despite continuous work to develop new drugs for cancer, there are still problems facing its treatment. Cancer occurs by tumor cell proliferation with interacting stromal component resulting in a compound tumor microenvironment (TME) [1]. Cancer is not just proliferating malignant neoplastic cells. It is an intricate mass of neoplastic cells along with stromal cells undergoing phenotypic changes that form tumor-associated stroma and actively participate in carcinogenesis [2]. Genomic alterations and inflammatory response by immune cells in the tumor stroma result in certain features characterizing cancer known as hallmarks of cancer. They are sustaining proliferation signals, evasion of growth inhibitory signals, evasion of apoptosis, replicative immortality, prompting angiogenesis, triggering invasion and metastasis, reprogramming of energy metabolism, and evasion of immune destruction [3]. Recently, new enabling characteristics and emerging hallmarks were added: unlocking phenotypic plasticity, non-mutational epigenetic reprogramming, polymorphic microbiomes, and senescent cells [4].

Cancer is a complex genetic disease that is not caused by mutation in just one gene. For instance, one molecular signaling pathway controlling cell growth is disturbed by many genetic aberrations [5]. Since the evolution of genomics, transcriptomics, proteomics, and metabolomics in the current century, the field of targeted therapy has developed. The production of drugs directed toward molecules driving carcinogenesis while minimizing off-target effects is the key of targeted therapy. Cancer targets include growth factors, growth factor receptors, signal transducing molecules, transcription factors, proapoptotic and antiapoptotic proteins [6]. These targets are abnormally expressed in cancer due to genetic mutations of oncogenes and tumor suppressor genes [7]. Genetic mutations can be corrected by using therapeutic nucleic acids (TNAs). The administration of genetic material into living cells to replace a defective gene, promote or repress gene expression to treat a disease is known as gene therapy [8].

MicroRNAs (miRNAs) are non-coding RNAs (ncRNAs) that regulate gene expression by acting on messenger RNA (mRNA). A miRNA binds the 3′ untranslated region (UTR) of a target mRNA to cause gene silencing. miRNAs control many cellular processes such as cell proliferation, differentiation, cell death, and metabolism [9]. The control starts from biogenesis of miRNAs where there are genes in the genome specialized for miRNAs, which are transcribed into primary miRNAs (pri-miRNAs) with stem loops. Pri-miRNA is cleaved by ribonuclease Drosha and its partner DGCR8 to give a shorter stem-loop structure: precursor miRNA (pre-miRNA), which is transported to the cytoplasm for maturation and performing action. The expression of miRNAs is altered in cancer which in turn disturbs the expression of oncogenes and tumor suppressor genes: the main genes driving cancer. miRNAs can be targeted in gene therapy to restore their normal levels [1].

In this review, we will summarize the principle of gene therapy and highlight the RNA interference (RNAi) natural process and method of applying cancer gene therapy. Then, we will elaborate the rising roles of miRNA in diagnosis and treatment of cancer and how to tackle the limitations of its application in treatment.

### Review search methodology

Online search on (PubMed, Google Scholar, ScienceDirect) databases was done to identify original research and review articles using the keywords and search terms: “Cancer”, “Targeted therapy”, “Gene therapy”, “Gene expression”, “epigenetic”, “gene therapy vectors”, “nanoparticles”, “antisense oligonucleotides”, “RNA interference”, “microRNAs”, “miRNA”, and “exosomes”. The search was conducted starting from March 2023 to December 2023 considering only English written articles.

### Targeted gene therapy

The aim of novel cancer therapies is to provide recovery of patient with increased survival rate and reduced recurrence rate along with preserving the surrounding healthy tissue minimizing as much as possible adverse side effects [10].

Targeted therapy is a mode of cancer therapy depending on administering drugs that target molecules uniquely or differentially expressed in cancer cells in comparison with normal cells. This targeting interferes with tumor cell growth and proliferation. The advantage is that it does not affect normal cells and thus avoiding toxic side effects. Targets can be growth factors, growth factor receptors, signaling molecules, and apoptotic molecules altered by genetic mutations or epigenetic dysregulation [5].

Most agents used for targeted therapy are biopharmaceuticals, which are drugs derived from biological sources. They are made of sugars, proteins, nucleic acids, or may be living cells like reproductive cells and stem cells [11].

Targeting genes can be done by administering nucleic acids into the cell to treat a disease through restoring the function of a mutated gene, regulating gene expression, or synthesizing a new protein. This is the concept of gene therapy. The introduced genetic material may be a gene, segment of a gene, or oligonucleotide and is referred to as transgene [12].

Gene therapy can be used in the treatment of various diseases ranging from inherited to acquired diseases. Many clinical trials have been applied on different diseases with promising results especially single gene disorders [8]. This encouraged researchers to exploit gene therapy for cancer treatment [12].

Gene therapy can be applied in cancer through targeting its molecular processes [1]. Target cells can be cancer cells, normal cells, or immune cells. For cancer cells, the transgene can kill them or regain normal function. While for normal cells, the transgene can shield them from toxicities of a chemotherapeutic drug. Immune cells can also be targeted to recognize and kill cancer cells. The transfer of the nucleic acids is carried out by one of two methods: ex vivo and in vivo. In the ex vivo method, the tumor cells are collected, grown in culture under controlled conditions, manipulated genetically, and inserted back into the host. The in vivo method is applied by introducing the transgene systemically or locally into the tumor cells [12].

There are different modalities for implementing cancer gene therapy such as replacement of tumor suppressor genes, transcription factor decoys, suicide genes, oncolytic viruses, gene editing, oncogene silencing, and targeting miRNAs. Other modalities focus on the TME including immunization gene therapy, targeting angiogenic molecules, cancer-associated fibroblasts, and tumor cell-derived exosomes [1]. The modality nominated for each patient depends on his/her genetic profiling, immune status, and nature of the tumor [12].

### RNA-based treatment

The flow of genetic information from DNA to RNA and from RNA to protein represents the central dogma of molecular biology. The RNA molecule transcribed from DNA and encoding a protein is called mRNA. Cell functions are performed by proteins. RNA molecules that do not give rise to proteins are called ncRNAs but they are involved, by different ways, in regulating gene expression, protein synthesis, and activity. They include ribosomal RNA, transfer RNA, long non-coding RNA, small interfering RNA (siRNA), miRNA, and others. Blocking the step of protein synthesis is the basis of RNAi [13].

Treatment-wise, targeting RNA offers many treatment options and techniques due to the different types of RNAs [13]. Also, acting on the level of RNA is safer than DNA to minimize the risk of mutation. Since RNA is unstable, its delivery into cells is more challenging than DNA where it is subjected to clearance by the kidneys and degradation by endogenous ribonucleases [14]. Chemical modifications are done to the synthetic RNA-based molecules to tackle these obstacles [13] and will be discussed later in this review.

RNA-based treatment can be achieved either through double-stranded (ds) RNA molecule or single-stranded (ss) antisense oligonucleotide (ASO). The dsRNA is converted by ribonucleases into ssRNA that is complementary to the target mRNA and degrade it. The ASO has several mechanisms of action depending on the binding to a specific sequence of the target mRNA: activating RNase H for degradation of mRNA, preventing 5′ cap formation, blocking protein translation, and altering splicing [13].

### RNA interference

RNAi is a physiological process used by the body innate immune system to fight viral infections and as a mechanism of regulating gene expression. mRNA function of coding proteins can be blocked through its degradation or inhibiting the translation process. The result is known as gene silencing. A group of ncRNAs act on mRNAs, the most common are siRNAs and miRNAs [13].

ncRNAs are among the epigenetic factors that control gene expression. Epigenetic modification is any event that affects gene expression without changing DNA sequence. DNA methylation, histone modifications, and ncRNAs are mechanisms of epigenetic modification. Interestingly, there is an interplay between different epigenetic mechanisms. ncRNAs can act on mRNAs that code for enzymes that perform DNA methylation and histone modification: DNA methyl transferase (DNMT) and histone acetyl transferase (HAT)/histone deacetylase (HDAC), respectively. These enzymes also act on promotors of DNA that is transcribed to ncRNAs [15].

One method of applying gene therapy for cancer is oncogene silencing which is based on the process of RNAi. This can be done by introducing a synthetic siRNA or short hairpin RNA (shRNA) that acts on the mRNA of an oncogene such as MYC or KRAS [1].

### MicroRNAs

miRNAs are short non-coding ss RNAs [16]. Their length is about 18–25 nucleotides. miRNAs function in regulation of gene expression after transcription by complementary binding to mRNAs [17]. Regulation of the target mRNA is done by one or more of the following processes: translation inhibition, deadenylation, decapping, and mRNA degradation by exonucleases [18]. Several biological processes are controlled by miRNAs such as cell division, differentiation, cell death, and metabolism [19].

The first discovered miRNA was miRNA lin-4. The lin-4 gene was detected in the nematode *Caenorhabditis elegans* (*C.elegans*). To the surprise, it did not code for a protein but rather two small RNA molecules ranging from 20 to 70 nucleotides. The longer was the precursor of the shorter. They have sequences complementary to a sequence at the 3′ UTR of lin-14 mRNA [20]. Moreover, mutations in lin-14 result in a phenotype contrasting that of lin-4 mutations and are completely dependent on them [21]. Later, miRNA let-7 was also discovered in *C. elegans*, and then additionally in multicellular organisms and animals [22]. At present, there are nearly 3000 identified miRNAs in human genomes regulating more than 30% of genes [18].

miRNAs are encoded from different regions in the genome. Intergenic regions are the commonest, followed by introns, exons, long non-coding regions, and repeat regions [18]. These regions are called cancer-associated genomic regions because they are liable to alterations in different cancers [23].

**Fig. 1:**
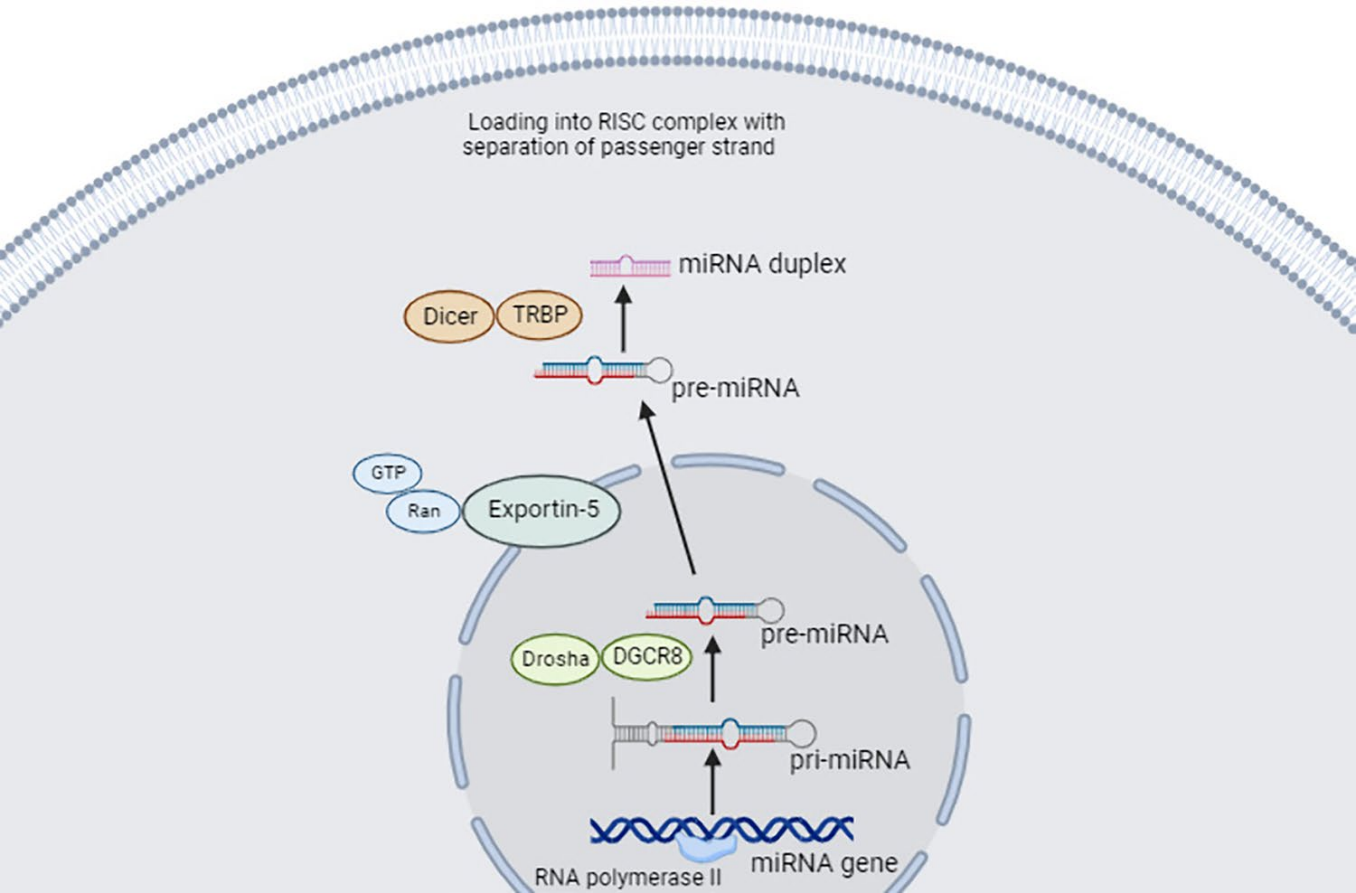
MicroRNA processing (Created with BioRender.com)

**Fig. 2:**
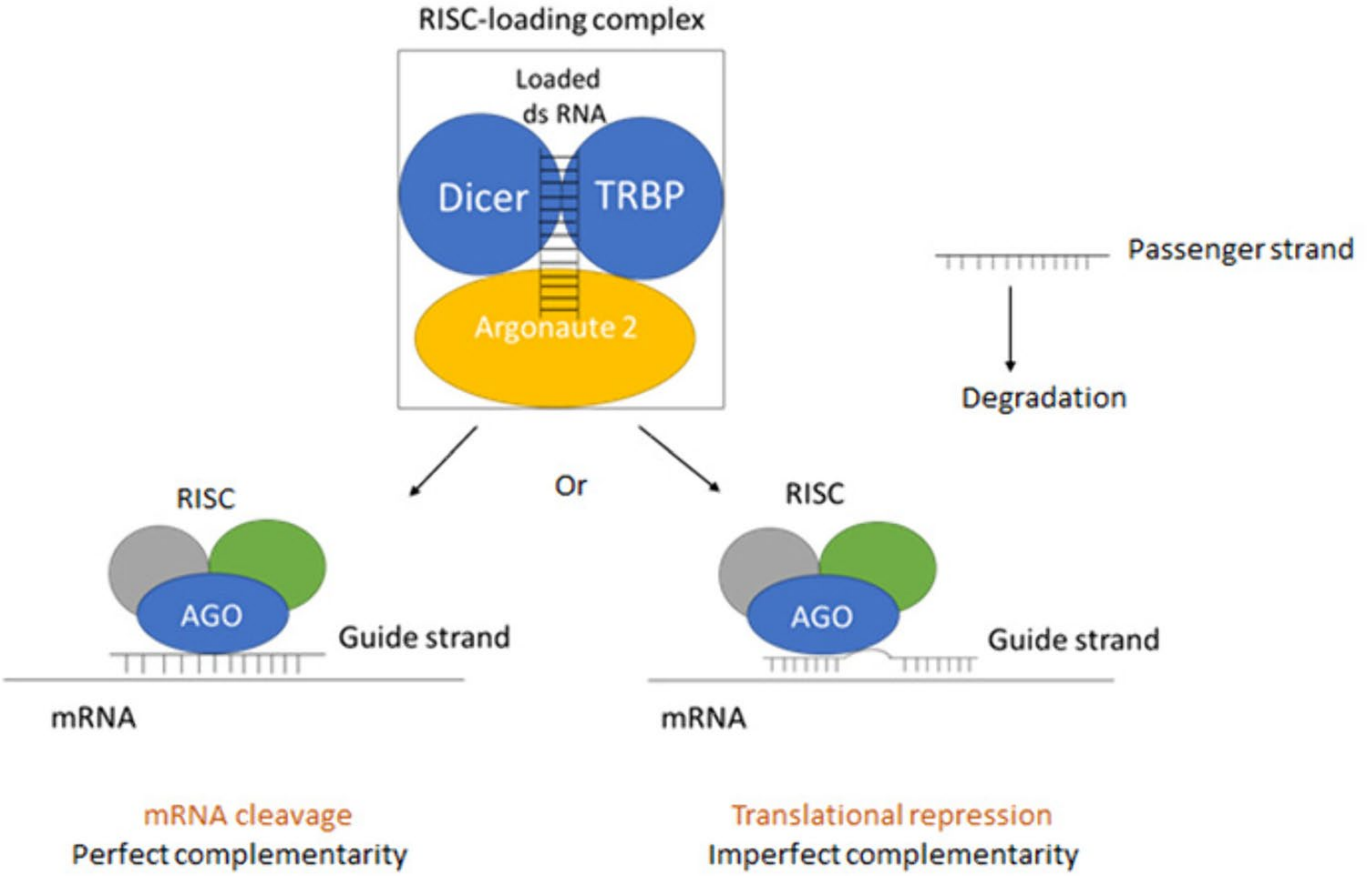
MicroRNA mechanism of action. Loading of double-stranded RNA into RNA-induced silencing complex (RISC) with separation of passenger strand that will be degraded. The guide strand along with Argonaute 2 protein and other proteins form active RISC complex that is directed to the target mRNA and acts on it. (Created with Microsoft PowerPoint)

Generation of miRNA starts by transcription from a specific sequence of DNA using RNA polymerase II to produce a pri-miRNA. pri-miRNA is a dsRNA with a stem loop and is cleaved by ribonuclease Drosha and its partner DGCR8 to give a pre-miRNA. pre-miRNA is transported to the cytoplasm through exportin-5 bound to Ran-GTP, where the stem loop is cut by ribonuclease Dicer/TRBP to give a miRNA duplex [24, 25] (Fig.1). The miRNA duplex is loaded into a multicomplex protein called RNA-induced silencing complex (RISC) with Argonaute 2 (AGO2) endonuclease protein as the active part. miRNA is separated into two strands: the guide stand (antisense strand) and the passenger strand (sense strand). The passenger strand is degraded while the guide strand within the RISC complex is directed to the target mRNA. Unlike siRNA, miRNA does not perfectly bind mRNA [26] (Fig.2). There is a specific sequence in the miRNA about 7 bases near the 5′-end that binds to a complementary sequence of the target mRNA at the 3′ UTR or less commonly 5′ UTR or coding region. It is called miRNA seed. Not all other miRNA bases match the mRNA bases [13]. Therefore, a single miRNA can target many mRNAs and an individual mRNA can be targeted by various miRNAs [27].

Apart from their standard role in regulation of gene expression through targeting mature mRNA, miRNAs have other targets. Examples are transcripts of introns and intergenic regions, other ncRNAs, circular RNAs, pseudogenes, and short interspersed nuclear elements [18]. This explains the indirect role of miRNAs in regulation of gene expression. Not all mature miRNAs function in the cytoplasm, some are transported back to the nucleus where they carry out other functions such as regulating the maturation of other miRNAs and regulating the expression and maturation of ncRNAs. Also, post-transcriptional regulation of pre-mRNAs in the nucleus can sometimes be done by miRNAs. Moreover, they can act on certain gene promoters causing transcriptional silencing via RNA-induced transcriptional silencing (RITS) complex [28]. Apart from nuclear and cytoplasmic miRNAs, some miRNAs are expressed in extracellular vesicles (ECVs), where they act on other cells [29]. Others are expressed in mitochondria and are involved in mitochondrial functions [30].

It was found that miRNAs regulate the functions of tumor suppressor genes and oncogenes. A miRNA acting on the mRNA of a tumor suppressor gene is classified as oncomiRNA since it inhibits its protective role against cancer. Likewise, a miRNA acting on the mRNA of an oncogene is classified as tumor suppressor miRNA [31]. Studies have found that miRNAs are involved in cellular processes that are disturbed in cancer such as cell proliferation, cell death, migration, and angiogenesis [32]. Oncogenesis does not only arise from genetic mutations and epigenetic alterations in protein-coding genes but also from similar events in miRNAs genes. Furthermore, defects in miRNA synthesis steps share in oncogenesis. For example, loss of function mutation in exportin-5 gene hinders the transport of pre-miRNAs from the nucleus to the cytoplasm and subsequently impairs their processing and functioning [33].

The expression of miRNAs is abnormal in cancerous tissues. miRNA genes are subjected to mutations similar to protein-coding genes such as amplification or deletion. Epigenetic change in the form of methylation of CpG islands of miRNA promoters is also common. In cancer, there is either upregulation or downregulation of miRNAs and it reflects the alterations noted in expression levels of oncogenes and tumor suppressor genes [16]. For example, miRNA-21 is overexpressed in non-small cell lung cancer (NSCLC) and inhibits the activity of PTEN: a tumor suppressor gene [34]. Likewise, miRNA-155 is highly expressed in breast cancer and it downregulates SOCS1 gene [35]. On the other hand, miRNA-29 plays a role in apoptosis and was found to be downregulated in hepatocellular carcinoma. A study conducted by Xiong et al. revealed that the low expression of miRNA-29 was inversely correlated with those of antiapoptotic genes Bcl-2 and Mcl-1 [36]. Another example of tumor suppressor miRNAs are miRNA-34 family members (a, b & c) that are deregulated in various cancers such as leukemia [37], colon cancer [38], prostate cancer [39], head and neck cancer [40], and oral cancer [41].

Some miRNAs can act as oncomiRNAs or tumor suppressor miRNAs in different occasions depending on the target mRNA, since one miRNA has many target mRNAs that can be involved in distinct cellular pathways [16].

Some miRNAs act on enzymes of epigenetic action such as DNMT and HDAC which in turn affect the expression of protein-coding genes. Abnormal expression of miRNAs results in atypical DNA methylation and histone modifications that are associated with oncogenesis. Hypermethylation or hypomethylation of miRNA promotor regions are also causes of aberrant miRNA expression [42]. A study conducted by Kozaki et al. found that miR-34a, miR-137, miR-193a, and miR-203 are downregulated in oral squamous cell carcinoma (OSCC) due to hypermethylated promotor regions [43].

An expression profiling study about OSCC ascertains that upregulated miRNAs are related to tumor suppressor genes, while downregulated miRNAs are related to oncogenes [23]. Decreased expression of miRNA-99A-5p was associated with increased expression of NOX4 in OSCC cells as deduced by Shi et al. [44]. In contrast, Peng and colleagues found that high expression level of miRNA-134 was associated with low expression level of PDCD7 in OSCC tissues [45]. Using bioinformatics tools for mapping functional pathways can reveal the pathways associated with deregulated miRNAs. For example, PI3K/Akt pathway involved in proliferation, migration, and apoptosis is activated in oral cancer and this activation was linked to miRNA-16 and miRNA family let-7, which are downregulated [23]. An experimental study on bladder cancer cell lines concluded the role of tumor suppressor miRNAs-143 and miRNAs-145 on PI3K/Akt signaling pathway [46].

Many miRNAs are involved in the carcinogenesis of cholangiocarcinoma (CCA) by their downstream effects on target genes [47]. For example, miRNA-194 is important in stemness through ECT2 gene [48], while miRNA-329, miRNA-144-5p, and miRNA-451 are important in proliferation and tumor growth through PTTG1 and ST8SIA4 genes, respectively [49, 50]. Moreover, miRNAs such as miRNA-7-5p, miRNA-137, miRNA34a, and miRNA-424-5p play vital roles in CCA progression by triggering invasion, migration, and metastasis. Interestingly, it was found that miRNAs promote CCA by controlling several signaling pathways linked to cancer such as PI3K/AKT, Wnt/β-catenin, NF-κB, p53, Notch, and Hedgehog signaling pathways [47].

Renal cell carcinoma (RCC), the most common cancer of the kidneys, is mediated by dysregulation in miRNAs. miRNAs orchestrate signaling pathways such as RAS/MAPK, Wnt/β-catenin, TGF-β/NF-κB, and VEGF signaling, and dysregulated miRNAs bring about disrupted actions of signaling molecules involved in these signaling pathways which are linked to RCC [51].

Angiogenesis is one of the key features of cancer. In order to survive, cancer cells need blood supply which is preserved through sustained angiogenesis. Like all types of cancer, laryngeal cancer is maintained by angiogenesis and miRNAs have a deep impact on the process [52]. Zhang et al. reported low expression of miRNA-140-5p in laryngeal cancer tissues and transfection of miRNA-140-5p-expressing plasmid in laryngeal cancer cell line inhibited proliferation and angiogenesis. There was negative correlation between miRNA-140-5p expression and VEGF-A expression, which is a main driver of angiogenesis [53].

miRNAs serve as valuable diagnostic and prognostic biomarkers for cancer [32]. Detecting the levels of miRNAs in body fluids can be used for early detection, diagnosis, and monitoring of treatment in different types of cancer [17, 32, 47, 51, 52, 54]. Unlike most RNA molecules in the extracellular environment which are degraded by ribonucleases, extracellular miRNAs detected in body fluids are stable [16]. This could be due to their package in ECVs [29], or loading into high-density lipoproteins [55], or being bound by AGO 2 proteins [56], which protect them from degradation [29]. miRNA profiling denotes detection of their levels in patient’s samples and can be done using laboratory methods including quantitative reverse transcription polymerase chain reaction (qRT-PCR), microarrays, and next-generation sequencing [32]. Ganepola et al. carried out microarray analysis for profiling miRNAs from blood samples that can help in diagnosing early stage of pancreatic cancer [57]. Furthermore, a study conducted by Zhu et al. observed, using qRT-PCR, a number miRNAs with significant higher expression in blood samples of lung cancer patients than healthy controls [58].

miRNAs also act as targets for therapy [23]. Instead of the conventional techniques of gene therapy such as targeting a coding gene or its mRNA, miRNA-based therapy is focused on targeting miRNAs correcting their expression levels. This will subsequently correct the underlying abnormal expression of mRNAs [1].

Treatment using miRNAs can be carried out through two approaches. The first one is based on restoring the function of downregulated tumor suppressor miRNAs by introducing a miRNA mimic. This method is known as miRNA replacement therapy. Transfection of miRNA-340 mimic in NSCLC cell lines reduced cell proliferation and prompted apoptosis as reported by Fernandez et al. [59].

The second treatment approach is inhibiting the function of an upregulated oncomiRNA by a miRNA inhibitor [17]. There are different forms of miRNA inhibitors such as anti-miRNA oligonucleotides (AMOs), miRNA sponges, and miRNA masks. AMO is an ASO that binds the miRNA of interest blocking its action [60]. Sharma and colleagues delivered anti-miRNA-191, loaded onto liposomes, in breast cancer cells, and resulted in induced apoptosis and reduced cell migration [61].

miRNA sponge is a plasmid that can be transfected into cells and expresses transcripts containing multiple miRNA binding sites that bind the target miRNA so that miRNA cannot bind mRNA. An advantage of miRNA sponge is that it can target a family of miRNAs that share the same seed sequence not just a single miRNA [62] as is the case of AMO which requires more sequence complementarity than just the seed region [63]. Expressions of miRNA-221 and miRNA-222 in OSCC cells were reduced when using miRNA-221/222 sponge, while expression of its target gene PTEN was increased. As a result, proliferation and invasion of OSCC cells were inhibited with induced apoptosis [64].

Another form of miRNA inhibitors is miRNA mask that competes with miRNA on the miRNA binding site of mRNA [60]. Other than targeting an oncogenic miRNA by hybridization, inhibition of miRNA activity can be done by interfering with the miRNA machinery. An example is molecules that deactivate dicer enzyme that cleaves pre-miRNA to mature miRNA [65].

An important hindrance related to chemotherapy is the development of drug resistance. Deep studying of the role of miRNAs in cancer has revealed that they are responsible for therapy-related resistance by controlling mechanisms such as drug efflux, DNA damage repair, epithelial–mesenchymal transition, apoptosis, and autophagy [66, 67]. From this point, exploiting deregulated miRNAs and correcting their expression help in improving treatment outcomes [17]. Restoration of normal miRNA expression levels in RCC helped in increased sensitivity to chemotherapeutic drugs. For instance, miRNA-30c-expressing lentiviral vectors were transfected in highly aggressive clear cell RCC cell line (Caki-1) and resulted in increased chemosensitivity to sorafenib and paclitaxel through downregulating MTA-1 [68]. On the other hand, inhibition of miRNA-21 in RCC cell lines enhanced chemosensitivity to paclitaxel, 5-fluorouracil, oxaliplatin, and dovitinib by reducing expression of multi-drug resistance genes: ABCC3–6, ABCC2–6, and ABCC3 and ABCC5 [69]. In the study of Jiang et al., miRNA-577 expression helped increase sensitivity of colorectal cancer cells to 5-fluorouracil by targeting HSP27 with resultant decreased cell proliferation [70]. Likewise, Chai et al. found that miRNA-101 increased chemosensitivity of liver cancer cells to cisplatin by inhibiting DNA‑dependent protein kinase catalytic subunit (DNA-PKcs) signaling pathway [71]. miRNAs also have an impact on radiotherapy as detected by Mao et al. in which miRNA-449a improved radiosensitivity of prostate cancer cells by acting on c-MYC [72].

Drawbacks of applying microRNAs in cancer therapy include effects in non-targeted tissues, moderate efficacy, and delayed response time. Another problem is competition between synthetic miRNA and endogenous ones in RISC which may hinder the intended therapeutic effect [17]. Also, exogenous miRNA is susceptible to degradation by ribonucleases in blood [73]. Another shortcoming related to miRNA therapeutics is their multiple targets. One miRNA can bind to more than one mRNA altering their function. Similarly, an mRNA can be targeted by several miRNAs. Therefore, the binding is less efficient [17]. There are bioinformatics software platforms that can predict targets of a certain miRNA, and tools for gene enrichment analysis that help identify the biological pathways that a miRNA is involved in and its interactions with different protein-coding genes. These tools can facilitate the selection of the appropriate miRNA–mRNA candidate for treatment and adjusting the effective drug dose [74].

**Fig. 3:**
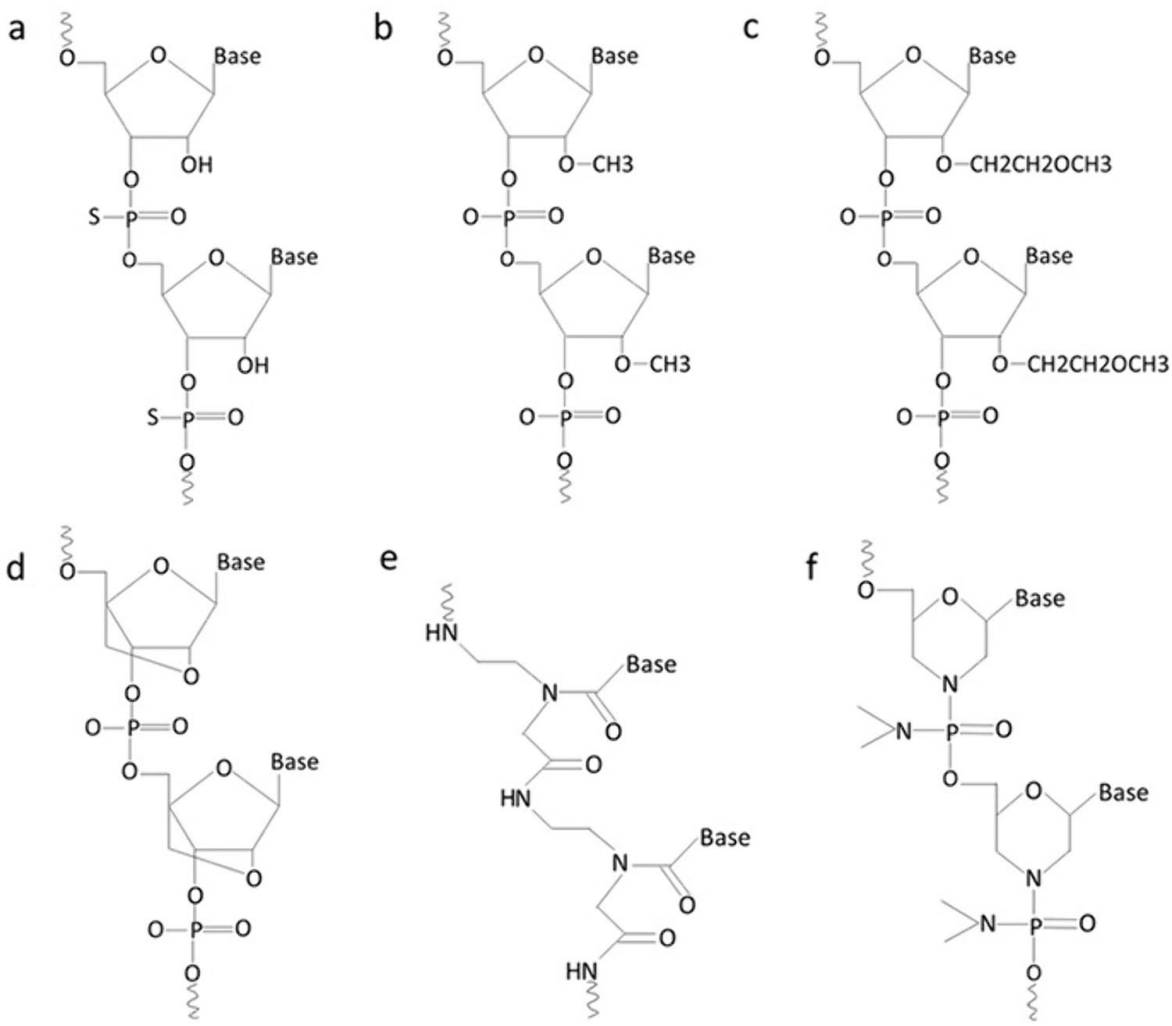
Chemical modifications of therapeutic RNA oligonucleotides. **a** Phosphorothioate RNA **b** 2′-O-Methyl RNA **c** 2′-O-Methoxyethyl RNA **d** Locked nucleic acid **e** Peptide nucleic acid **f** Phosphorodiamidate morpholino oligomer (Created with Microsoft PowerPoint)

Chemical modifications to exogenous miRNA therapeutic structures are done to improve their stability, specificity, and delivery and to decrease off-target effects. Modifications are in the form of changes made to the phosphodiester bonds, or sugar unit [13] (Fig.3).

Phosphorothioate (PS) bond is a modification by which non-bridging oxygen in the phosphodiester bond is replaced by sulfur atom making the link between nucleotides resistant to degradation by nucleases [75], allowing adequate RNase H activation for the target RNA degradation, and binding to plasma proteins mostly albumin that prevents loss of drug through renal infiltration and assists uptake by the target tissue [76]. An additional chemical modification of ASO with PS bond is done to the sugar unit by adding a methyl group to the hydroxyl group at 2 ′ carbon (2′-O-Me) or adding methoxyethyl (2′-O-MOE). This increases the binding stability to target RNA, decreases off-target effects, and increases half-life [60].

Locked nucleic acid (LNA) is a chemical structure in which 2′ oxygen and 4′ carbon of the ribose sugar of the synthetic RNA nucleotides are linked together. This increases the stability and binding affinity [77]. Obad et al. designed LNAs that can target the miRNA seed for optimal inhibition [19]. LNA-miRNA-361-3p was transfected into xenograft model of OSCC and resulted in reduced tumor size. Using qRT-PCR after transfection, the expression of miRNA-361-3p in tumor tissue was reduced, while the expression of its target gene OSR2 was elevated [78].

Peptide nucleic acid (PNA) and phosphorodiamidate morpholino oligomer (PMO) are other structures with sugar-phosphate modifications [76]. They work by blocking translation but do not activate H RNase activity [13]. PNA is a neutral synthetic oligonucleotide in which the sugar phosphodiester backbone has been altered with N (2-amino ethyl)-glycine unit [60]. It is more stable than true oligonucleotides encouraging its use as an antisense gene in gene therapy. It binds with high specificity to target sequence [79]. PMO is also a neutral synthetic oligonucleotide in which ribose rings are replaced with hexagonal morpholine rings and phosphodiester bonds are replaced with phosphorodiamidate linkages [80]. Like PNA, PMO is stable, resistant to degradation by endogenous nucleases and possesses specificity for binding target sequence. Additionally, it is water soluble [81].

In addition to chemical modification of therapeutic miRNA molecules, they can also be conjugated with small transport domains like cell penetrating peptides (CPPs) and aptamers to improve delivery. CPPs are short peptides with sequences less than 40 amino acids. They are positively charged and can bind to their cargo by covalent or non-covalent bonds [82]. They transfer across the cell membrane by electrostatic interaction with plasma membrane constituents followed by direct translocation or endocytosis depending on the size of the cargo [83]. CPPs are suitable for delivery of neutral charged oligonucleotides like PNA and PMO [84]. Modifications are done to the structure of CPPs to make them capable of endosomal escape and releasing their cargo into the cytosol [85]. Besides, CPPs can be combined with nanoparticles improving delivery and anti-tumor effectiveness [83].

**Fig. 4:**
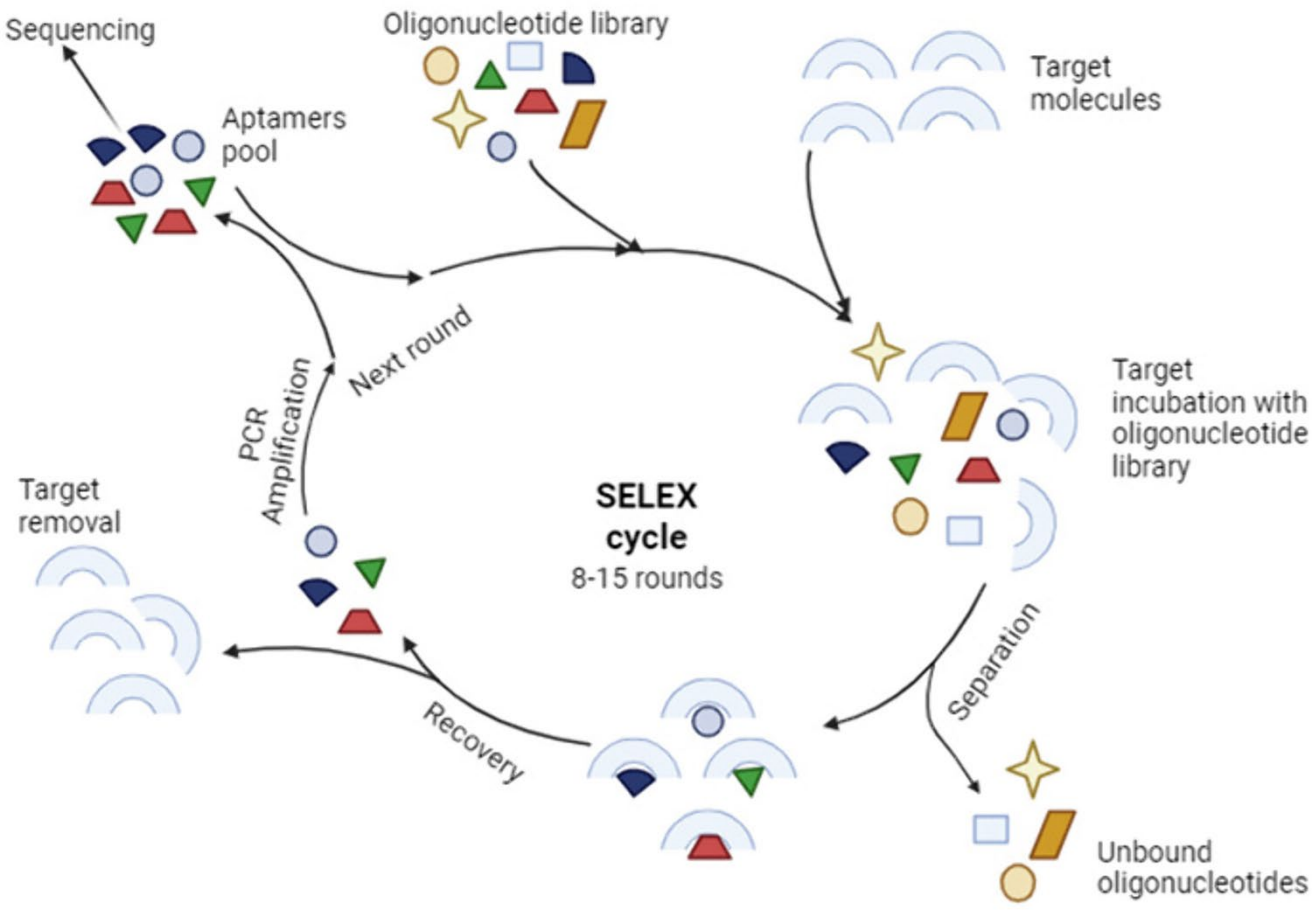
Selection and synthesis of aptamers by systematic evolution of ligands by exponential enrichment (SELEX) (Created with BioRender.com)

Aptamers are single-stranded oligonucleotides that can bind to their target molecules with high affinity and specificity since they are generated by systematic evolution of ligands by exponential enrichment (SELEX) (Fig.4). Aptamers are named chemical antibodies but they are much more stable due to their nucleic acid composition. Besides, they are less immunogenic, non-toxic, and can penetrate target tissue more easily due to their smaller size. They can directly deliver TNAs such as siRNA and miRNA or they can become conjugated with nanoparticles carrying TNAs. In both ways, the aptamer acts as a ligand binding a cell surface target (receptor) and internalize into the cell by receptor-mediated endocytosis [86].

Introducing TNAs such as miRNA into the target cells is not an easy task, since there are many barriers faced during the process of delivery such as passage through the cell membrane, nuclease activity, immune response, and blood clearance. Thus, an effective and safe vehicle (vector) is crucial to ensure success of gene therapy [1].

### Vectors for gene therapy

There are two types of vectors: viral and non-viral vectors [55]. Viral vectors are the initial early vehicle suggested for gene therapy. They are characterized by their transfection efficiency. Yet, immune reaction and cancer risk related to their use induced the innovation of non-viral vectors [1].

Viruses bind to target cells and introduce their genetic materials inside the cells as they undergo replication. The transfer of genetic material by a viral vector is called transduction. Different viruses are genetically engineered to be used as vectors. There are lentiviruses, adenoviruses, and adeno-associated viruses. Lentiviruses are RNA viruses and have the ability to integrate their genetic material into the host genome by reverse transcription, while adenoviruses and adeno-associated viruses are DNA viruses and exist in extrachromosomal location (episome) [12]. Lentiviruses are characterized by high transfection efficiency, good compatibility, and low immunogenicity [87]. However, they have the risk of developing second malignant neoplasms due to their integration into the host genome [88]. Adenoviruses also have high transfection efficiency. In addition, their transgene packaging capacity is large. But they result in transient expression of the target gene and elicit a strong immunogenic response. Adeno-associated viruses are less immunogenic than adenoviruses and offer more prolonged gene expression [12] yet, it has limited transgene capacity [87]. Another DNA viral vector is herpes simplex virus that has great transgene capacity, ability of transducing a wide variety of cells, and low immunogenicity thanks to genetic engineering [12].

Non-viral vectors include lipid-based (liposomes & solid lipid nanoparticles), polymer-based, or inorganic nanoparticles [1]. Gene therapy established by non-viral vectors is known as transfection. Non-viral vectors have lower transfection efficiency than viral ones but they are safer and can be easily modified [12]. They are also cost effective and can be produced on a large scale [89].

Nanoparticles are particles whose size ranges between 1 and 100 nm. Materials at these small sizes acquire different physical and chemical properties than at macroscopic sizes that can be tailored to interact with biological molecules and cells [1]. Nanoparticles are a safe alternative to viral vectors owing to their biocompatibility and low immunogenicity [89]. One important feature of nanoparticles as a carrier for any cancer therapeutic drug not only TNAs is increasing the concentration of drug at the tumor site. This will make use of inhibition of tumor cells along with decreased toxicity on the surrounding healthy cells [90].

Different carriers of nanosize are available for delivery of cancer therapeutic agents. There are inorganic nanoparticles, polymeric nanoparticles, and lipid nanoparticles [90].

Inorganic nanoparticles are made from metals, silica, or carbon. Metallic nanoparticles, specifically gold derived, are commonly used in nanomedicine. Gold nanoparticles can be easily manipulated into different structures enhancing biocompatibility and incorporation of drugs. Their optical properties help tracking its location intracellularly [1]. Gold nanoparticles are favored for use in gene therapy due to increased cellular uptake, rapid endosomal escape, and adequate half-life [91]. Silica and carbon nanoparticles have the advantage of large surface area for functionalization; however, their toxicity is a major drawback limiting their use [1].

Compared to inorganic nanoparticles, organic nanoparticles have lower toxicity on biological systems. They are either lipid-based or polymer-based. Lipid nanoparticles are characterized by an amphiphilic nature, which makes them the best match with cell membrane interaction and offers the delivery of various molecules [92]. There are two forms of lipid nanoparticles: liposomes and solid lipid nanoparticles. They differ mainly in the nature of the core whether aqueous or lipidic, and the number of lipid layers [1]. Polymeric nanoparticles have good biocompatibility, biodegradation, and controlled release in target cells. They are derived from natural compounds or are synthesized [93]. Polymeric nanoparticles are positively charged so they can easily interact with nucleic acids which carry negative charge. Despite these favorable properties, they are less effective than other nanoparticles for delivery of genetic material. Some authors developed modifications to improve their quality such as coating polymeric nanoparticles with polyethylene glycol (PEG) that enhanced the transport through biological barriers [93, 94]. Also, conjugation of polyethyleneimine (PEI) nanoparticles with ECVs improved the delivery of small RNAs in various cell lines [95].

ECVs are membrane bound particles secreted by eukaryotic cells and are responsible for cell–cell communication. They are also secreted by prokaryotes. Many molecules are transferred by ECVs such as lipids, proteins and nucleic acids. ECVs regulate normal physiological processes and have a role in the pathology of many diseases. They promote tumorigenesis by inducing cell proliferation, angiogenesis and matrix remodeling. Being identified in body fluids through specific markers, ECVs serve as valuable diagnostic tools [96]. According to the way of vesicular secretion from cells, ECVs are classified into microvesicles and exosomes [29]. Microvesicles are directly shed from the cell membrane and their sizes range from 50 nm up to 1000 nm. Exosomes are derived from endolysosomal pathway that generate multivesicular bodies. Exosomes are synthesized by inward budding of a multivesicular body membrane. They are released from cells by exocytosis when multivesicular body fuse with the plasma membrane [96]. Their size ranges from 40 nm up to 200 nm [95]. There is more focus on exosomes in studies or they are referred to generally as ECVs [29, 95].

Exosomes can deliver different molecules such as proteins, lipids, and nucleic acids between cells providing a way of intercellular signaling. Various functions are done by the communication between cells including proliferation, differentiation, antigen presentation, tissue repair, and healing [71]. Exosomes extracted from cancer patients express biological markers that can aid in diagnosis and early detection [29]. Moreover, they can be used as vehicles to deliver therapeutic agents including TNAs like miRNA into diseased cells [73]. Since exosomes can be derived from the patients from the appropriate cells, they are biocompatible, and does not elicit an immunogenic response [96]. They are also stable ensuring efficient delivery of miRNAs into the target cells [73]. Another advantage of using exosomes is that they transport their payload directly into the cytosol so the process of endosomal escape is not required [95]. Exosomes proved success in delivery of miRNAs to cancer cells in experimental studies such as miRNA-159 and-497 in triple-negative breast cancer and NSCLC, respectively [97, 98]. However, poor loading efficiency and difficulty in exosomes characterization and purification owing to their heterogeneity impede their use [99].

### Stem cell vectors

Stem cells are promising vehicles for cancer gene therapy. Their tumor tropism nature makes them a good choice for delivery of suicide genes, anti-tumor miRNAs, and viruses [100].

Mesenchymal stem cells (MSCs) can be isolated from various tissues such as bone marrow, adipose tissue, and umbilical cord. MSCs do not activate the immune system due to low or absence of expression of major histocompatibility complex class I and class II [101]. Therefore, they can be introduced into recipients without human leukocytic antigen matching. A feature of MSCs is homing toward damaged tissues, inflamed tissues, and tumors. Homing to tumor site facilitates their use as vehicles for delivery of anticancer drugs including TNAs. Beside their ability to migrate to primary tumor sites after intravenous administration, MSCs can migrate toward metastatic tumor sites. The TME is rich in inflammatory cells that secrete inflammatory cytokines, and also tumor cells secrete growth factors. These secreted products chemically attract MSCs. Of great and specific importance is tumor necrosis factor alpha (TNF-α): a cytokine that stimulates the expression of vascular cell adhesion molecule-1 (VCAM-1) through NF-κB signaling pathway. VCAM-1 directs the adherence of MSCs to endothelial cells of tumor blood vessels which helps penetration and accumulation of MSCs at tumor sites [102].

Transduction of the transgene into MSCs is established by viral or non-viral vectors [101]. Interestingly, exosomes isolated from MSCs represent a superior delivery vehicle for cancer gene therapy. MSCs-derived exosomes have the same advantages of MSCs with elimination of any immune response and with no risk of differentiation capacity [103]. MSCs-derived exosomes loaded with miRNAs or anti miRNAs proved success in reducing tumor cell proliferation, invasion, and metastasis in a variety of cancer types through studies performed on cell lines and xenograft models [104–106]. Efficient correction of miRNA expression with corresponding target gene is achieved.

### Monitoring drug uptake and response

Uptake of therapeutic agents into the cells in in vitro studies requires evaluation to ensure the first step of successful treatment and afterward, implementation in vivo. Visualization can be done by fluorescent microscope where the transgene is fluorescently labeled or by electron microscope [99].

The effect of molecularly targeted drug including TNAs on tumor tissue can be evaluated using functional imaging. Positron emission tomography (PET) scan utilizing fluorodeoxyglucose (FDG) radioactive tracer measures metabolism of glucose which is higher in cancer cells in comparison to normal cells. FDG is taken up by cancer cells and in case of response to treatment, the uptake decreases [5].

### Current and future outlines

Detection of miRNAs in body fluids served in diagnosis and monitoring of many types of cancer such as lymphomas and cancers of lung, liver, breast, stomach, colon, and pancreas [107]. The field of miRNA therapeutics is still evolving. Although there is a huge number of preclinical studies applied on different types of cancer with promising results, ongoing clinical trials are modest and have not reached phase III. Some trials have stopped due to adverse side effects. Delivery vehicles, routes of administration, dosage, effects in non-target tissues, and cost are still hindering the translation of miRNA therapeutics to the clinic and approval of drugs for patients [9]. More efforts are recommended to transfer basic research to the clinic [17].

For optimum treatment results, gene therapy can be combined with another treatment option like chemotherapy, radiotherapy, or immunotherapy. Correction of gene problem improves the response from the adjuvant treatment [12]. Additionally, drug delivery utilizing the nanotechnology helps better distribution of the drug at the tumor tissue and minimizes deleterious side effects on normal tissues [90]. Polymeric nanoparticles such as dendrimers were effectively used as a vehicle for combined delivery of miRNA-21 inhibitor and the chemotherapeutic drug doxorubicin into breast cancer cells with good antitumor effect [108].

Undergoing genetic analysis before cancer treatment is a promising procedure that can guide the choice of the most appropriate treatment for the patient, protecting him/her from the prolonged duration of inappropriate treatment choices [31]. The ultimate aim is to save the patient the good way without badly influencing his/her life.

## Conclusions

The study of miRNAs has revealed their importance in physiological processes and pathological conditions such as cancer. miRNAs are reliable molecular markers reflecting presence of the disease, its stage, and its response to treatment since they are differentially expressed between normal and diseased tissues. They are also stable in body fluids providing a non-invasive tool for screening. Targeting miRNAs is a promising treatment for cancer that needs to be translated to clinics hopefully after dealing successfully with its obstacles. The use of nanotechnology for miRNA delivery into cells can make the best of benefitting from the miRNA wide impact on gene regulation, and resolving the genetic complexity of cancer.

## Data Availability

All data generated or analyzed during this study are included in the manuscript.

## Abbreviations

AGO2: Argonaute 2
AMO: Anti-miRNA oligonucleotide
ASO: Antisense oligonucleotide
CCA: Cholangiocarcinoma
*C.elegans*: Caenorhabditis elegans
CPP: Cell penetrating peptide
DNMT: DNA methyl transferase
ds: Double-stranded
ECVs: Extracellular vesicles
FDG: Fluorodeoxyglucose
HAT: Histone acetyl transferase
HDAC: Histone deacetylase
LNA: Locked nucleic acid
Me: Methyl
miRNA: MicroRNA
MOE: Methoxyethyl
MSCs: Mesenchymal stem cells
ncRNA: Non-coding RNA
NSCLC: Non-small cell lung cancer
OSCC: Oral squamous cell carcinoma
PEG: Polyethylene glycol
PEI: Polyethyleneimine
PET: Positron emission tomography
PMO: Phosphorodiamidate morpholino oligomer
PNA: Peptide nucleic acid
PS: Phosphorothioate
pre-miRNA: Precursor miRNA
pri-miRNA: Primary miRNA
qRT-PCR: Quantitative reverse transcription polymerase chain reaction
RCC: Renal cell carcinoma
RISC: RNA-induced silencing complex
RITS: RNA-induced transcriptional silencing
RNAi: RNA interference
SELEX: Systematic evolution of ligands by exponential enrichment
shRNA: Short hairpin RNA
siRNA: Small interfering RNA
ss: Single-stranded
TME: Tumor microenvironment
TNAs: Therapeutic nucleic acids
TNF-α: Tumor necrosis factor alpha
UTR: Untranslated region
VCAM-1: Vascular cell adhesion molecule-1
